# Is root canal treatment and an indirect coronal restoration of a mandibular first molar cost-effective compared to extraction and an implant-supported crown? A decision analytic approach

**DOI:** 10.2340/aos.v84.42894

**Published:** 2025-02-27

**Authors:** Nikki Savolainen, Fredrik Frisk, Thomas Kvist

**Affiliations:** a Public Dental Services, Region Jönköping, Sweden; b Department of Endodontology, Institute for Postgraduate Dental Education, Jönköping, Sweden; c School of Health and Welfare, Jönköping University, Jönköping, Sweden; d Department of Endodontology, Institute of Odontology, The Sahlgrenska Academy, University of Gothenburg, Gothenburg, Sweden

**Keywords:** Cost-effectiveness, decision analysis, root canal treatment, indirect coronal restoration, implant-supported single crown

## Abstract

**Objective:**

The aim was to compare the cost-effectiveness of root canal treatment and an indirect restoration (RCT + PC) versus an implant-supported single crown (ISSC) in the case of a decayed first mandibular molar with a necrotic pulp. The study was based on Swedish population and the reference prices for dental treatments in Sweden.

**Materials and methods:**

The cost-effectiveness for RCT + PC and ISSC were calculated with the help of two decision trees. The initial costs were from the Swedish dental reference prices in 2024, and the probability values were from published articles based on Swedish data.

**Results:**

In Decision tree I, the expected costs for RCT + PC and for ISSC were 17,400 and 18,800 SEK, respectively. In the Decision tree II, the respected expected costs were 19,500 SEK and 18,800 SEK. The threshold probability values were 83 and 93% for RCT survival.

**Conclusions:**

Given the assumptions and limitations of this decision analysis, the probability of survival for RCT + PC needs to be in the range of 83–93% in order for it to be more cost-effective than ISSC, when deciding about treatment on a compromised first mandibular molar.

## Introduction

Oral health in Sweden has improved significantly during the last few decades. Edentulism and partial edentulism are less common than before [1]. Missing teeth affect negatively the oral health-related quality of life [2] and is also associated with a higher risk for cardiovascular diseases [3]. Therefore, in case of a severely decayed tooth with pulp necrosis, today’s patients may not accept tooth extraction. At the same time, it is not considered adequate to retain a compromised tooth without considering other treatment options.

In the case of a tooth with a necrotic pulp, root canal treatment (RCT) is needed before restoration with an indirect post and core (PC). Guidelines from the *Swedish National Board of Health and Welfare* recommend RCT due to the low costs in relation to gained effect [4]. Indeed, this treatment has been shown to perform well, with survival rates reaching 86–93% [5, 6]. However, even though the development of treatment techniques and materials [7] have been remarkable, RCT is a technically challenging treatment [8, 9]. In one survey conducted in Gothenburg, Sweden, general dental practitioners expressed a lack of sufficient expertise when performing RCT [10]. In a later prospective study of RCT in the very same organization [11], it was noted that 22.1% of initiated RCTs in molars ended up with the tooth being extracted before treatment was completed with a permanent root filling. The data suggest a need for a reconsideration of clinicians’ preoperative tooth survival estimation before initiating RCT.

Instead of doing RCT and PC on a severely decayed tooth with a necrotic pulp, there is the alternative to extract the tooth and replace it. If the nearby teeth are intact, guidelines from the *Swedish National Board of Health and Welfare* recommend an implant-supported single crown (ISSC) [4]. Implant-supported prosthetic constructions have become more common among patients in Sweden [1], and systematic reviews have shown ISSCs to have high survival rates [12]. However, the treatment has its biological and technical complications [12, 13], and loss of the implants does occur. In such a situation, the final solution to replace a tooth is to take advantage of neighboring teeth as abutments for a fixed dental prosthetics (FDP) [4].

The high survival rates of ISSC can make this treatment seem superior to RCT and PC, but it needs to be noted that the high initial costs [14, 15] can be too high for some patients. In maxillary incisors, it has been shown that RCT is more cost-effective than ISSC [16]. However, the circumstances for RCT differ between tooth types; hence it should be avoided to directly generalize the results of Pennington et al. 2009 [16]. For example, the number of root canals and the complexity of RCT are making the molars more expensive to treat compared to incisors.

Clinical decision analysis has its roots in ‘expected utility theory’ (EUT) by Neumann and Morgenstern [17]. It was presented for medical decision analysis in 1959 by Ledley and Lusted [18] and has thereafter been used, for example, in cost-effectiveness analyses [19], including dentistry [20]. The problem being solved is first turned into a model, for example, a decision tree. The model visualizes the different outcomes the situation may have. When the decision is to be based on cost-effectiveness, the outcomes include as factors both costs and probabilities. The information for these factors can be obtained in different ways, but the probabilities are usually from published high-quality scientific literature [21]. Together the costs and probabilities can be used to calculate expected costs for the outcomes, and the one with the lowest expected cost is considered to be the most cost-effective. To show the threshold values for when one option becomes more cost-effective than the other, a sensitivity analysis is used. It shows how much the variables can be changed before the other alternative becomes more cost-effective.

This study aims to compare, by a decision analytic approach, the cost-effectiveness of RCT and PC versus ISSC in the situation of a severely decayed first mandibular molar with a necrotic pulp in the context of Swedish general dental practice.

## Materials and methods

This is a model-based cost-effectiveness study. No patients or patient data were used, and hence, no external ethical approval was needed. Current available data on outcomes based on Swedish data were preferred when available so that conditions and assumptions were cohesive and valid within this selected population. Costs were elicited from the reference pricelist established by *the Swedish Dental and Pharmaceutical Benefits Agency (TLV)* [14, 15].

### Decision tree model

**Figure 1:**
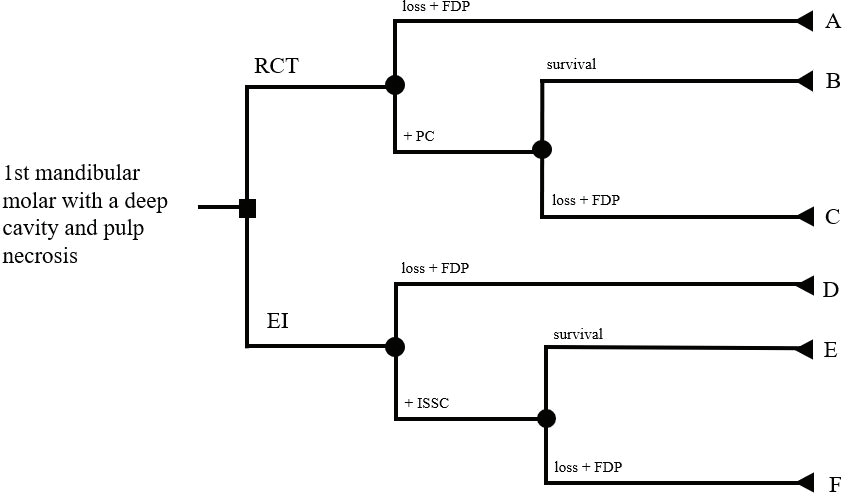
Decision tree I. If a tooth or an implant is lost, a fixed dental prosthesis is chosen.

**Figure 2:**
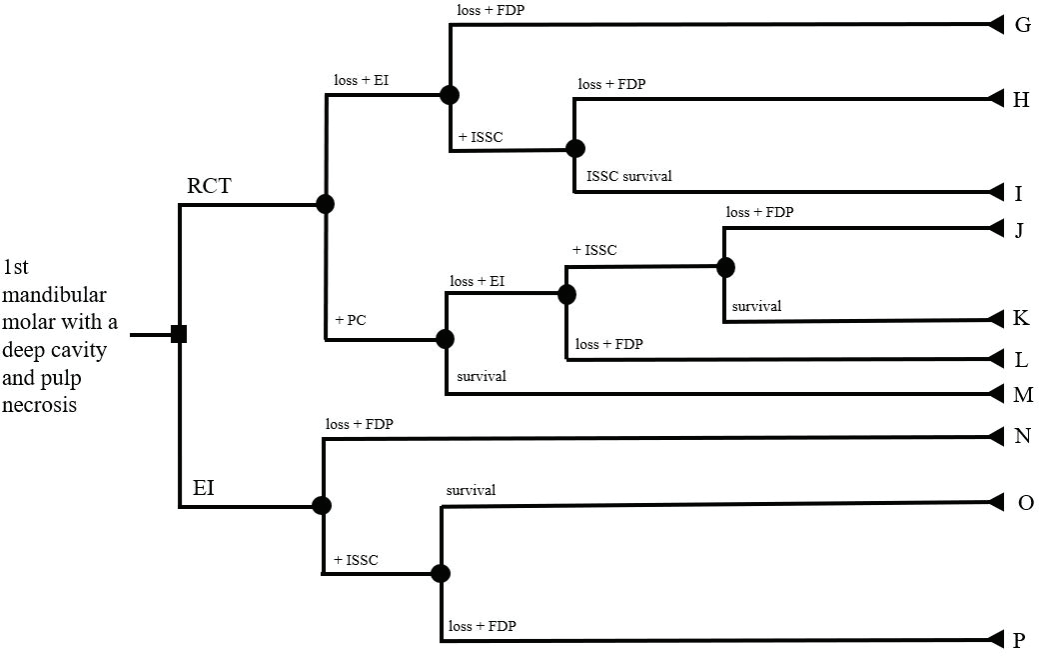
Decision tree II. If an implant is lost, a fixed dental prosthesis is chosen. If a tooth is lost, an implant installation is first made, and in case of a loss of the implant, a fixed dental prosthesis is chosen.

This study used a decision tree to visualize the treatment scenarios. Two different decision trees were made, see Figures 1 and 2. The starting point in both decision trees is identical; a first mandibular molar with a deep caries lesion and a dental pulp necrosis, leading to two available treatment options (the first branching). The first treatment option is an RCT, and the intention of a PC. The second option is extraction of the tooth and installing an implant (EI), to be finished with an ISSC. In the decision trees, applying a new treatment step is an active choice, while survival or loss of a tooth or an implant is given by probabilities. The outcome is controlled at two different time points after the treatment choice: 1 year and 5 years postoperatively. Each branch with a single endpoint is denoted by an uppercase letter (A to P). Assumptions for the decision-tree setup were:

- The patient does not accept a tooth gap in the position of a first mandibular molar
- The patient has no medical or health contraindications
- The patient presents with a full set of teeth
- The patient has no other dental problems
- No other dental treatment is being planned
- No maintenance or reparation is needed during the follow-up period
- No noble metals are used in the prosthetic treatments
- The treatments are performed at a general dental practice clinic except for the surgical procedures relating to the implant treatment

#### Decision tree I

In the case of choosing RCT, the outcome after 1 year is either failure to finalize RCT, followed by extraction and an FDP (A), or tooth survival and placement of a PC. In the case of a PC, the tooth survives for the full follow-up period (B), or the tooth is extracted during the follow-up period followed by an FDP (C).

In the case of choosing EI, the outcome after 1 year is either a failed implant installation followed by an FDP (D), or a successful implant installation followed by an ISSC. In the case of ISSC, it either survives for the follow-up period (E) or is lost and replaced by an FDP (F).

#### Decision tree II

In Decision tree II, after choosing an RCT but failing to finalize it, an implant is installed. This implant can then be lost and replaced by an FDP (G). If the implant survives and an ISSC is placed, it can either be lost and replaced with an FDP (H) or survive the follow-up period (I).

If the tooth survives during the RCT, it is followed by the placement of a PC, which can be lost followed by EI and an ISSC. This in turn can be lost followed by an FDP (J) or survive (K). The EI can also be lost before an ISSC and hence an FDP is placed (L). RCT followed by a PC can also result in survival for the full follow-up period (M).

In the Decision tree II, the outcomes for the EI branches are identical to the ones in Decision tree I: the implant can be lost at the second branching in the decision tree and replaced with an FDP (N). If an ISSC is made, it can either survive at the third branching in the decision tree (O) or be lost and replaced with an FDP (P).

### Probabilities (P-values)

Probabilities for the branches in the decision trees were derived from published articles on tooth and implant survival and losses in the context of Swedish general dentistry. Assumptions and approximations were needed when eliciting probability values:

- If the study did not specify the number of mandibular molars out of all molars, it was assumed that all the molars in the study were mandibular molars
- If the study did not make a difference between different types of coronal restorations, it would be assumed that teeth with a root filling had a PC
- If no difference was made between different implant positions, it was assumed that all implant positions had the same survival
- If no difference was made between the different implant-supported prosthetic constructions, it was assumed that all constructions had the same survival

#### Estimation of probability values in Decision tree I

The probability value for tooth loss during the first year after RCT, P(A), was elicited from Wigsten et al. [11] according to the following formula: $P(A)=\frac{extracted\;molars}{all\;molars}$. The survival during first year was then $1-P(A)=1-\frac{extracted\;molars}{all\;molars}$.

The probability values of branches B and C were calculated in two steps. In the first step, the study by Fransson et al. [22] was utilized. Then, the numbers from step 1 were multiplied with the 1-year follow-up information calculated with the formula used above.

Step 1: $\begin{array}{c} P(RCT\;step\;1)\;\;=\;\frac{mandibular\;molars}{all\;teeth}\;\\ \times \;survival\;of\;PC\;\\ +\;\frac{teeth\;except\;mandibular\;molars}{all\;teeth}\\ \;\times \;survival\;of\;mandibular\;molars \end{array}$

Step 2:

$\begin{array}{c} P(B\;)=\;P(RCT\;step\;1)\;\times \;(1\;–\;P(A\;))\;=(\frac{mandibular\;molars}{all\;teeth}\;\\ \;\times \;survival\;of\;PC\text{+ }\frac{teeth\;except\;mandibular\;molars}{all\;teeth}\\ \times \;survival\;of\;mandibular\;molars)\;\\ \;\times \;\left(\;1\;–\;\;\;\frac{extracted\;molars}{all\;molars}\right)\;.\\ \; \end{array}$

Since P(A), P(B), and P(C) together equaled 1: *P(C) = 1 – P(A) – P(B).*

The 1-year follow-up probability values for the EI branches, P(D) and 1 – P(D), were calculated using data from Derks et al. [23] with the following formulas: *P(D) =*

$\frac{implants\;lost\;before\;the\;prosthetic\;construction\;was\;made}{all\;implants}$, and

$1-P(D)=1-\frac{\begin{array}{l} implants\;lost\;before\;the\;prosthetic\;\\ construction\;was\;made \end{array}}{all\;implants}.$

The same way as in calculating for RCT, the 5-year follow-up values for EI branches were calculated in two steps. The values utilized in the calculations’ first step were from Cecchinato et al. [24]. This way, the probability values for branches E and F were calculated as follows:

Step 1: $\begin{array}{c} P\left(EI\;step\;1\right)\;=\;\;\frac{implants\;lost}{all\;implants}\;.\\ 1\;–\;P\left(EI\;step\;1\right)\;=1\;–\frac{implants\;lost}{all\;implants}. \end{array}$

Step 2: $\begin{array}{l} P(E)=(1-P(D))\times (1-P(EI\;step\;1))\\ \;=\;\left(\;1\;–\frac{\begin{array}{l} implants\;lost\;before\;the\;prosthetic\;\\ construction\;was\;made \end{array}}{all\;implants}\right)\\ \;\times \;\left(\;1–\;\frac{implants\;lost}{all\;implants}\right)\;. \end{array}$

Since P(D), P(E), and P(F) together equaled 1: *P(F) = 1 – P(D) – P(E)*. The calculations for Decision tree I can be seen in Table 1.

**Table 1:** Calculations for probability values (P-values) in Decision tree I. The P values were given with 2 decimals. Since the sum of outcome P-values in each treatment group needs to equal 1, some adjustments needed to be made. P(C) was rounded to 0.08 since this was smaller rounding compared to if the same was done for P(A) or P(B). In case of EI, the P(E) was rounded to 0.98, since this way the proportions between the different P(EI) did not change drastically.

Outcome	Calculation	*P*	*P* (2 decimals)
A	$\frac{25}{113}$	0.2212389…	0.22
B	$\left[0.254\times \;\left(1\;-\;\frac{914}{20\;308}\right)+\left(1-0.254\right)\;\times \;\left(1\;-\;\frac{6237}{55\;173}\right)\right]\times \left(1\;–\frac{25}{113}\right)$	0.7041846…	0.70
C	$1-\;\frac{25}{113}\;-\;\left\{\left[0.254\times \left(1\;-\;\frac{914}{20\;308}\right)+\left(1-0.254\right)\times \left(1\;-\;\frac{6237}{55\;173}\right)\right]\;\times \left(1\;–\frac{25}{113}\right)\right\}$	0.0745764…	0.08
D	$\frac{154+26}{11311+2367}$	0.0131598…	0.01
E	$\left(1-\frac{154+26}{11311+2367})\times (1-\frac{11}{729}\right)$	0.9719495…	0.98
F	$1–\frac{154+26}{11311+2367}\;-\text{ [(1}-\frac{154+26}{11311+2367})\times (1-\frac{11}{729})]$	0.0148905…	0.01

#### Estimation of probability values in Decision tree II

Since the branches of EI were the same in Decision I and II: P(D) = P(N), P(E) = P(O), and P(F) = P(P).

The probability value calculations of RCT outcomes in Decision tree II were based on the probability values of branches A, B, and C, as well as D, E, and F. These previously calculated probability values were multiplied with each other in different combinations, based on the components of each outcome: All the probability values with RCT loss used P(A). In case of EI loss, P(D) was one of the multipliers. When an ISSC survived, P(E) was used. If an ISSC was lost, P(F) was used instead. In the branches where a PC was lost, P(C) was a multiplier. By following this flow, the following formulas were made:

*P(G) = P(A) × P(D),*

*P(H) = P(A) × P(F),*

*P(I) = P(A) × P(E),*

*P(J) = P(C) × P(F),*

*P(K) = P(C) × P(E),*

*P(L) = P(C) × P(D),*

*P(M) = P(B).*

These calculations are exhibited in Table 2.

**Table 2:** Calculation for probability values (P-values) in Decision tree II. The P-values were given with 2 decimals. Since the sum outcome P-values in each treatment group need to equal 1, some adjustments needed to be made. Since with rounding to 2 decimals and with the condition that no P < 0.01, the sum of the rounded P-values was 0.03 too high. To avoid this, 0.01 was taken from the rounded P-values that were higher than 0.01.

Outcome	Calculation	*P*	*P* (2 decimals)
G	$\frac{25}{113}\;\times \;\frac{154+26}{11311+2367}$	0.0029114…	0.01
H	$\frac{25}{113}\;\times \;\left\{1\;\;–\;\frac{154+26}{11311+2367}\;–\;\left[\left(1\;–\frac{154+26}{11311+2367}\;\right)\;\times \;\left(1\;-\;\frac{11}{729}\right)\right]\right\}$	0.0038235…	0.01
I	$\frac{25}{113}\;\times \;\left[\left(1\;–\frac{154+26}{11311+2367}\right)\;\times \;\left(1\;-\;\frac{11}{729}\right)\right]$	0.2150330…	0.21
J	$\begin{array}{l} \left\{1–\frac{25}{113}\;–\left\{\left[0.254\times \;\left(1\;-\;\frac{914}{20\;308}\right)\;+\left(1-0.254\right)\;\times \;\left(1\;-\;\frac{6237}{55\;173}\right)\right]\;\times \;\left(1\;–\frac{25}{113}\right)\right\}\right\}\\ \times \left\{1\;–\frac{154+26}{11311+2367}\;–\;\left[\left(1\;–\frac{154+26}{11311+2367}\right)\;\times \;\left(1\;-\;\frac{11}{729}\right)\right]\right\}\; \end{array}$	0.0012888…	0.01
K	$\begin{array}{l} \left\{1–\frac{25}{113}\;–\left\{\left[0.254\times \;\left(1\;-\;\frac{914}{20\;308}\right)\;+\left(1-0.254\right)\;\times \;\left(1\;-\;\frac{6237}{55\;173}\right)\right]\;\times \;\left(1\;–\frac{25}{113}\right)\right\}\right\}\\ \times \;\left[\left(1\;–\frac{154+26}{11311+2367}\right)\;\times \;\left(1\;-\;\frac{11}{729}\right)\right]\; \end{array}$	0.0734511…	0.06
L	$\left\{1\;–\;\frac{25}{113}\;–\;\left\{\left[0.254\times \left(1\;-\;\frac{914}{20\;308}\right)+\left(1-0.254\right)\;\times \;\left(1\;-\;\frac{6237}{55\;173}\right)\right]\;\times \;\left(1\;–\;\frac{25}{113}\right)\right\}\right\}\;\times \;\frac{154+26}{11311+2367}$	0.0009814…	0.01
M	$\left[0.254\times \;\left(1\;-\;\frac{914}{20\;308}\right)+\left(1-0.254\right)\;\times \;\left(1\;-\;\frac{6237}{55\;173}\right)\right]\;\times \;\left(1\;–\frac{25}{113}\right)$	0.7041846…	0.69

### Treatment costs

The costs of each branch were mainly elicited from *the TLV* reference price lists on different dental treatments in 2024 [14, 15] (see Table 3). No clinical or radiographic examination costs were included, since it can vary which examinations are needed in each case. For the hourly costs rate of a general practitioner, the price used in Västra Götaland county in 2024 [14] was used since it has no reference price given by *TLV*. When calculating the cost of RCT that was lost during the first year, it was assumed that 1 h of treatment time had been used before the decision to extract the tooth. When the tooth needed to be extracted, it was assumed that separation of the roots was needed since this is often the case with permanent molars. The cost of tooth extraction was included in all treatments where a tooth was lost. No maintenance costs were included. When an implant was removed during the first year, it was assumed that the removal did not cost any extra for the patient. When an implant was removed later, it was assumed that it was done surgically. An FDP was supposed to constitute two abutments and one pontic.

**Table 3:** Combinations of treatment number codes by *Swedish Dental and Pharmaceutical Benefits Agency* that were used in the calculations of costs.

Outcome	Treatment code combination by TLV
A	VG911 + 402 + 801 + 801 + 804
B or M	503 + 802 + 800
C	503 + 802 + 800 + 402 + 801 + 801 + 804
G	VG911 + 402 + 421S + 420 + 422S + 801 + 801 + 804
H	VG911 + 402 + 421S + 420 + 422S + 850 + 429S + 801 + 801 + 804
I	VG911 + 402 + 421S + 420 + 422S + 850
J	503 + 802 + 800 + 402 + 421S + 420 + 422S + 850 + 429S + 801 + 801 + 804
K	503 + 802 + 800 + 402 + 421S + 420 + 422S + 850
L	503 + 802 + 800 + 402 + 421S + 420 + 422S + 801 + 801 + 804
D or N	421S + 420 + 422S + 801 + 801 + 804
E or O	421S + 420 + 422S + 850
F or P	421S + 420 + 422S + 850 + 429S + 801 + 801 + 804

TLV: Swedish Dental and Pharmaceutical Benefits Agency

### Expected treatment costs of RCT and ISSC

**Figure 3:**
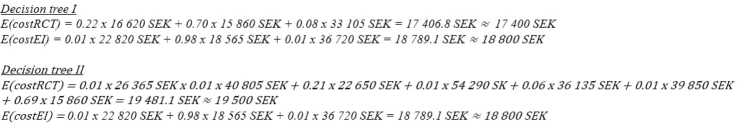
Calculations for expected costs in Decision trees I and II. The answers were rounded to hundreds.

The expected cost, E(cost), for RCT in Decision tree I was calculated with the following formula: *E(costRCT) = P(A) × Cost(A) + P(B) × Cost(B) + P(C) × Cost(C)*. For the EI, the same was calculated using the formula *E(costEI) = P(D) × Cost(D) + P(E) × Cost(E) + P(F) × Cost(F)*. The E(cost) for respective branches of RCT and EI in Decision tree II were calculated following the same systematic approach. The calculations are shown in Figure 3.

### Sensitivity analysis

**Figure 4:**
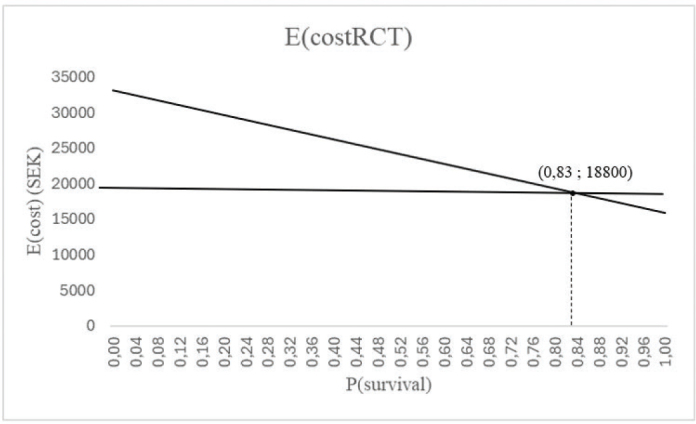
Sensitivity analysis for Decision tree I. When the P(survivalEI) is 0.98, the P(survivalRCT) needs to be at least 0.83 for root canal treatment + PC to be more cost-effective than ISSC. ISSC: implant-supported single crown; PC: post and core.

**Figure 5:**
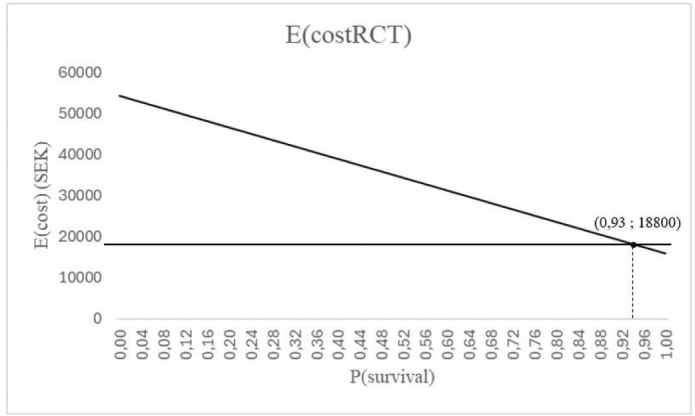
Sensitivity analysis for Decision tree II. When the P(survivalEI) is 0.98, the P(survivalRCT) needs to be at least 0.93 for root canal treatment + PC to be more cost-effective than ISSC. ISSC: implant-supported single crown; PC: post and core.

The one-way sensitivity analyses visualized, how much the probability value could be changed, that is, the threshold probability value, before one treatment option would become more cost-effective than the other. The two decision trees had their own sensitivity analyses, made by using Microsoft Excel (Microsoft^®^ Excel^®^ för Microsoft 365 MSO (Version 2312 Build 16.0.17126.20132) 64-bit edition), see Figures 4 and 5. In the sensitivity analyses, the probability for RCT and PC survival (branch B in Decision tree I and branch M in Decision tree II), representing the intended treatment choosing RCT, was altered from 0 to 100% on the x-axis, while the expected costs were on the y-axis. The E(costRCT) was shown as a diagonal line. The thresholds were then identified by marking E(costEI) at a certain probability value as a horizontal line, that cut the diagonal line at the threshold probability value.

## Results

### Decision tree I

The probability of 5-year survival of RCT with PC is 70% (B), and the corresponding probability for EI and ISSC is 98% (E) (Table 1). The cost for the different branches after choosing RCT varies between 15,860 SEK (B) and 33,105 SEK (C) (Table 4). The expected cost for choosing RCT is 17,400 SEK while the expected costs for choosing EI are 18,800 SEK (Figure 2). The probabilities, costs, and expected costs are summarized in Table 4.

**Table 4:** Probability values (*P* values), costs and expected costs of outcomes in Decision tree I.

Branch	Outcome	*P* (2 decimals)	Cost (SEK)	Expected cost (SEK)
A	RCT loss + FDP	0.22	16,620	3,700
B	RCT + PC survival	0.70	15,860	11,100
C	RCT + PC loss + FDP	0.08	33,105	2,600
RCT total		1.00		17,400
D	Implant loss + FDP	0.01	22,820	200
E	ISSC survival	0.98	18,565	18,200
F	ISSC loss + FDP	0.01	36,720	400
EI total		1.00		18,800

RCT: root canal treatment; ISSC: implant-supported single crown; PC: post and core; FDP: fixed dental prosthetics; EI: extraction and installing an implant.

### Decision tree II

No zero probabilities were allowed; hence, probabilities lower than 0.01 were rounded to 0.01, and consequently the probability of 5-year survival of RCT with PC is 69% (M), while the corresponding probability for EI and ISSC is identical to that in Decision tree I, 98% (O) (Table 2). The RCT branch costs vary from 15,860 SEK (M) to 54,290 SEK (J) (Table 5). The expected costs for choosing RCT are 19,500 SEK while the expected costs for choosing EI are the same as in Decision tree I, 18,800 SEK (Figure 3). The probabilities, costs, and expected costs for Decision tree II are summarized in Table 5.

**Table 5:** Probability values (*P* values), costs and expected costs of outcomes in Decision tree II.

Branch	Outcome	*P* (2 decimals)	Cost (SEK)	Expected cost (SEK)
G	RCT loss + EI + implant loss + FDP	0.01	26,365	300
H	RCT loss + EI + ISSC loss + FDP	0.01	40,805	400
I	RCT loss + EI + ISSC survival	0.21	22,650	4,800
J	RCT + PC loss + EI + ISSC loss + FDP	0.01	54,290	500
K	RCT + PC loss + EI + ISSC survival	0.06	36,135	2,200
L	RCT + PC loss + EI + implant loss + FDP	0.01	39,850	400
M	RCT + PC survival	0.69	15,860	10,900
RCT total		1.00		19,500
N	Implant loss + FDP	0.01	22,820	200
O	ISSC survival	0.98	18,565	18,200
P	ISSC loss + FDP	0.01	36,720	400
EI total		1.00		18,800

RCT: root canal treatment; ISSC: implant-supported single crown; PC: post and core; FDP: fixed dental prosthetics; EI: extraction and installing an implant.

### Sensitivity analyses

As in Decision tree I, the lowest estimated cost for choosing RCT would be 15,860 SEK ( = the probability of survival after restoration with a PC being 1), while the highest expected cost would be 33,105 SEK ( = the probability of survival after restoration with a PC being 0). Therefore, all the possible expected costs of RCT were placed on the line between these two points (Figure 4). The expected cost of ISSC in this study was 18,800 SEK, and in order to RCT to cost less than that, the probability value for RCT would need to be at least 83% (Figure 4).

As in Decision tree II, the lowest cost for RCT would still be 15,860 SEK, but the highest cost would be 54,290 SEK. In order to RCT to be more cost-effective than an ISSC in this scenario, the probability value of RCT survival would need to be at least 93% (Figure 5).

## Discussion

This cost-effectiveness analysis is a model analysis; hence, it gives a simplified version of reality in order to shed light on the cost-effectiveness differences between choosing RCT with the intention to restore with a PC or to extract a first mandibular molar in order to place an ISSC.

The analysis shows that based on the current state of evidence regarding the outcome of RCTs and dental implants, they are almost equivalent alternatives from a cost-effectiveness perspective, in the given clinical decision-making situation. Even a small deterioration in the pre-, intra-, and postoperative conditions for RCT and PC and thus its prognosis may change the expected cost and shift to favoring extraction and ISSC being more cost-effective treatment. In addition, the two sensitivity analyses show how the different plans on replacing a possible loss of the RCT tooth, that is, an FDP in Decision tree I and an ISSC in Decision tree II, affect the decision. This is illustrated by the fact that in Decision tree I, the analysis speaks in favor of starting RCT (lowest expected cost), while Decision tree II results show the reverse (lowest expected cost for choosing EI and an ISSC). It is important to point out the limitations and simplifications that allow the model to be considered as a basis for in-depth and refined analyses. We have assumed that the only valuable effect of the treatment is that there should be no tooth gap. We have thus assumed that a root canal treated tooth with a crown and an ISSC have the same instrumental value for this purpose. We have thus not weighed in the possible values in terms of aesthetics, chewing ability and the possible intrinsic value of being able to ‘keep one’s own teeth’. The decision can be affected by, for example, positions in the dental arch. In this study, the tooth in question was a first mandibular molar, which is not considered to be in the aesthetic zone, making the aesthetics less of a focus here.

The decision analysis model in our study is a variant of ‘EUT’ [25]. Although EUT has been questioned both as a normative and a descriptive theory [26], it indicates two essential components of a basis for making clinical decisions: the probability of something happening and the estimated value (utility) of that outcome. In our analyses we let cost act as a surrogate for utility value [27].

In our decision trees, we assumed that after RCT an indirect PC was needed since it has been shown that a PC has a positive impact on the survival of a root-filled tooth in case of severe tooth substance loss [24]. This assumption also seems most realistic from a clinical point of view since it is mainly when a large amount of coronal substance is missing that the question occurs whether it is better to extract the tooth and place an ISSC. However, many times a root-filled tooth is reconstructed with a direct coronal restoration [22], a factor with a potential effect both on the probability of survival as well as on cost and consequently expected cost. In this context, we underline that the probability values we used in our decision trees are estimates and interpretations of values taken from a few sources. For example, in Fransson et al. 2021 [22], it was not specified the number of molars that had an indirect restoration, and how many of these indirect restorations on molars were a PC. Therefore, a weighted average was used, where the survival of a tooth with a PC and survival of just mandibular molars in general, was used. The aim was to be able to show the better prognosis with a PC, but at the same time not to directly use a probability value for non-molar teeth. A modification on the type of supragingival construction was also made on ISSC, when it was chosen to have a screw-retained ISSC instead of a cemented one. This choice did not affect the initial costs since there are no different reference prices for screw-retained and cemented ISSCs, but it was made in order to get as similar ISSCs as possible, since the type of retention can affect, for example, the type of later complications [28]. The study by Cecchinato et al. [24] did not specify the prognosis of the different types of implant constructions, so it was assumed that the probability value for all the supragingival constructions was the same. This might have led to a slightly lower probability value than in reality, since it was stated in the article that the more extensive constructions had more complications [24]. In our study, we also did not take into account that implant survival rates may vary between different positions in the jaw because no such data are available in the current Swedish published articles. However, unlike with RCT where the number of root canals affects the cost, the position in the jaw does not affect the initial costs of the implant.

The estimated costs used for the treatments were the initial costs for ISSC and RCT + PC according to the 2024 version of the *TLV*’s reference price list [14, 15]. These reference prices are decided by the *TLV*, and they aim to guide the pricing for the different dental treatments in Sweden [29]. The prices in the reference list are based on the *TLV* panel of dentists and officials’ estimations of the actual costs for the procedures. It has been shown that these reference prices correspond well to the actual prices used by general dental practitioners [30], even though clinics and general organizations are allowed to decide their own pricelists [29]. Obviously, if the fees for the various procedures are set differently, the results of the expected costs will be different. In the sensitivity analyses (Figures 4 and 5), it is possible to extrapolate the position of the breaking point between RCT and ISSC by, for example, increasing and decreasing the costs of RCT and ISSC.

The reference price list includes even costs for different types of maintenance and repair [14, 15]. Complications and thus the need for maintenance treatment can be of both technical and biological nature for both an RCT + PC and an ISSC. Some can be relatively simple and therefore not particularly costly to treat. Others may be more complicated or require multiple visits.

With implants, technical complications can sometimes be solved by changing a part of the construction, but the complications in the soft tissues surrounding the implant can be more complicated [12, 31]. The peri-implant mucosa is not attached to the implant the same way as gingiva is to a tooth [32], affecting the need for maintenance. If, for example, the peri-implant mucosa starts to retract, it not only affects negatively the aesthetics by making the metallic parts visible, but it also creates new possibilities for plaque retention [32]. This makes it more difficult for the patient to perform optimal oral hygiene procedures and thereby increases the risks for peri-implant pathology, which brings more costs to the maintenance as well as affects the prognosis.

One main issue regarding maintenance of RCT + PC is that many root-filled teeth in Sweden, like in most countries, present with signs of persistent apical periodontitis [8]. In the mandibular molar region, the prevalence can be expected to be approximately 46% of the teeth [8]. Given that such a condition is in accordance with the current endodontic paradigm of what should be considered an endodontic ‘failure’ thus also requires some form of intervention, that is ortho- or retrograde revision treatment, the cost-effectiveness of initiating an RCT would be significantly impaired. However, it should be noted that a secondary treatment for, for example, a root-filled tooth does not automatically mean maintenance with a weak prognosis. While maintenance costs can be high, for example, apical surgery for a root-filled tooth costs 5,320 SEK according to the reference price list of special dental practitioners [15], it also prolongs the survival of the tooth, meaning that turning to FDP for a tooth replacement is not needed.

In this study, costs of any maintenance treatments were not included since there is a large variation of possible complications with RCT + PC and ISSC [33, 13], and these complications can be treated in different ways [33, 34]. However, this study can be used as a basis for such cost-effectiveness analyses. Further empirical research needs to be done to investigate what the actual costs, including maintenance costs, actually are for the various options. Best of all, this could be done in a randomized clinical trial where the two alternatives are set in opposition against each other in an adequate population.

It is important to also remember the other possible perspectives in the decision-making. For the patient, the real costs of the treatment are perhaps not just the cost paid to the dental clinic, but also if they must take time off from work, and how much the transportation would cost. Both ISSC and RCT, especially with an indirect restoration, take multiple appointments, and this can be something that affects the decision-making. There are also things not measured in money such as pain and fear. With an ISSC, the treatment is going to include surgery, which can cause certain emotions in patients. At the same time, even though RCT does not include surgery when done in an orthograde way, it can cause pain. A study by Torabinejad et al. [35] showed, however, that the difference between pain reception in these two treatments was small or nonexistent in a study population. For some patients, the importance of trying to retain their own teeth instead of replacing them may be of such great value that it trumps both cost and low probability of tooth survival. For the clinicians, there are different clinical factors that can weigh in at different rates, for example, systemic diseases, bone quality, and existing apical periodontitis [36].

Since the population of Sweden is getting older [37], dental treatments face new challenges. There are changes that are often associated with aging [38], such as decreased oral clearance and motoric skills, which can affect both the risk for dental caries as well as marginal periodontitis, if the oral hygiene is affected negatively. The forces directed to the prosthetic constructions can change. The survival and need of maintenance treatments are likely to be affected by this shift. It is also of interest to see, how the cost-effectiveness differences look like, when the follow-up period stretches over several decades instead of five years.

Lastly, it should be noted that the results of this study are based on Swedish studies as well as costs and economics in Swedish dental care. The results cannot be directly universalized, as different countries have different systems for financing dental care, and there may be different techniques used in RCT and ISSC, which could affect the probability values.

## Conclusions

Given the assumptions and limitations that apply to our analysis of how to decide about treatment on a compromised first mandibular molar, the probability of tooth survival after RCT and an indirect restoration needs to be in the range of 83–93% to be more cost-effective than extraction and an ISSC.
